# Simultaneous Bilateral Video-Assisted Thoracoscopic Surgery in the Prone Position for Resection of a Large Middle Mediastinal Tumor: A Case Report

**DOI:** 10.7759/cureus.79029

**Published:** 2025-02-14

**Authors:** Ryohei Miyazaki, Masaya Tamura, Marino Yamamoto, Hiroyuki Kitagawa, Satoru Seo

**Affiliations:** 1 Department of Thoracic Surgery, Kochi Medical School, Kochi, JPN; 2 Department of Surgery, Kochi Medical School, Kochi, JPN

**Keywords:** schwannoma, simultaneous bilateral approach, the middle mediastinum tumor, the prone position, vats

## Abstract

Schwannoma is the most common type of neurogenic tumor in the thorax and is typically benign. This article describes the case of a 64-year-old asymptomatic woman with abnormal findings on a chest X-ray examination. Preoperative enhanced computed tomography showed the presence of a middle mediastinal tumor measuring 7.4 cm protruding into both thoracic cavities. The mass was adjacent to the posterior aspect of the trachea. The esophagus was compressed to the right, indicating the potential appearance of symptoms in the future.

Consequently, we performed simultaneous bilateral video-assisted thoracoscopic surgery in the prone position, achieving complete resection. Postoperative pathological examination revealed a benign schwannoma. Here, we report a case of a middle mediastinal tumor resected by a rarely utilized video-assisted thoracoscopic surgery (VATS) method.

## Introduction

Schwannomas account for approximately 20% of mediastinal neurogenic tumors and are commonly found in individuals in their 20s and 30s. Schwannomas arising in the middle mediastinum are relatively rare, and there are various surgical approaches for resection of middle mediastinal tumors, depending on their location and size [1]. The selection of the approach is often challenging, as the presence of a middle mediastinal lesion and a large tumor can make removal difficult with a unidirectional approach and may require intraoperative repositioning. In this report, we describe a case in which a large schwannoma, arising in the middle mediastinum and protruding into both thoracic cavities, was resected using a bilateral video-assisted thoracoscopic surgery (VATS) approach in the prone position. This is the first report of this approach for such a tumor, and we discuss the usefulness, challenges, and limitations of this technique in this article.

## Case presentation

A 64-year-old female patient presented with a mass lesion detected on chest X-ray examination. Her medical history included hypertension, dyslipidemia, and surgery for a gastrointestinal stromal tumor of the small intestine performed shortly before presentation. Physical examination did not reveal remarkable findings. Preoperative blood analyses including tumor markers as well as urinary and respiratory tests yielded results within normal limits.

**Figure 1:**
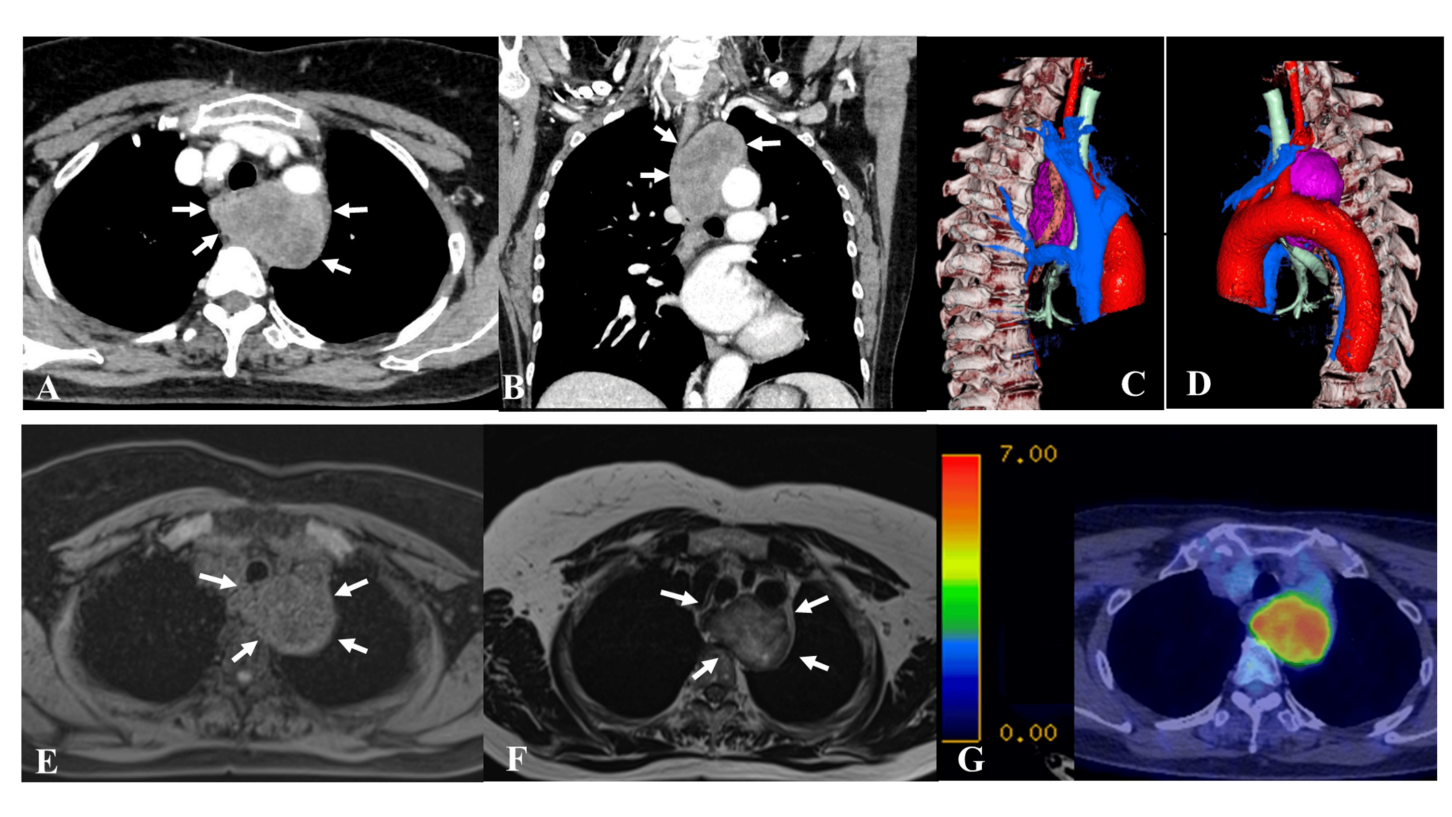
Radiological examination Chest-enhanced CT (A, B) and 3D-CT (C, D) showed a mass (diameter: 7.4 cm) located close to the posterior of the trachea with a clear border and heterogeneous contrast effect. The tumor was in contact with the medial side of the aorta, the dorsal side of the left subclavian artery, and the azygos venous arch, and severely compressed the esophagus to the right. Chest contrast MRI showed that the mass was not continuous with the esophagus and showed low T1 (E), heterogeneous high T2, and a low capsular signal on the periphery (F). On FDG-PET (G), the mass showed FDG accumulation with SUV_max_ of 8.2. 3D: Three-dimensional; CT: Computed tomography; FDG: 18F-fluorodeoxyglucose; MRI: Magnetic resonance imaging; PET: Positron emission tomography; SUV_max_: Maximum standard unit value.

Chest X-ray examination showed the presence of a mass lesion at the aortic arch. Chest-enhanced computed tomography (CECT) showed a mass (7.4 cm in size) with a clear border and heterogeneous contrast effect, located posterior to the trachea (Figure 1, Panels A and B). The mass abutted the inner surface of the aorta, the dorsal side of the left subclavian artery, and the azygos venous arch, and severely compressed the esophagus to the right (Figure 1, Panels C and D). Chest contrast magnetic resonance imaging demonstrated that the mass was not continuous with the esophagus and showed low signal intensity (SI) on T1-weighted images (WI) and heterogeneous high SI on T2-WI, with a low signal capsule at the edge on T2-WI (Figure 1, Panels E and F). Based on these findings, we suspected the mass to be a neurogenic tumor. However, positron emission tomography-CT (PET-CT) showed that the mass had 18F-fluorodeoxyglucose (FDG) accumulation with a maximum standard unit value of 8.2 (Figure 1, Panel G).

Based on this accumulation, the possibility of malignancy could not be ruled out. Malignant lymphoma, Castleman’s disease, and esophageal gastrointestinal stromal tumor were also considered in the differential diagnosis. Thus, endobronchial ultrasound-guided transbronchial needle aspiration and endoscopic ultrasound-guided fine needle aspiration were performed; nonetheless, these techniques did not lead to a definitive diagnosis. At that time, no signs of invasion were observed in the mucosa of the trachea or esophagus.

Despite the lack of a definitive diagnosis, we decided to perform surgical resection because of the possibility of respiratory distress due to tracheal compression in the future. After convening an interdepartmental meeting of the concerned specialties involved with the surgery, we selected bilateral simultaneous VATS in the prone position as the most appropriate surgical method.

**Figure 2:**
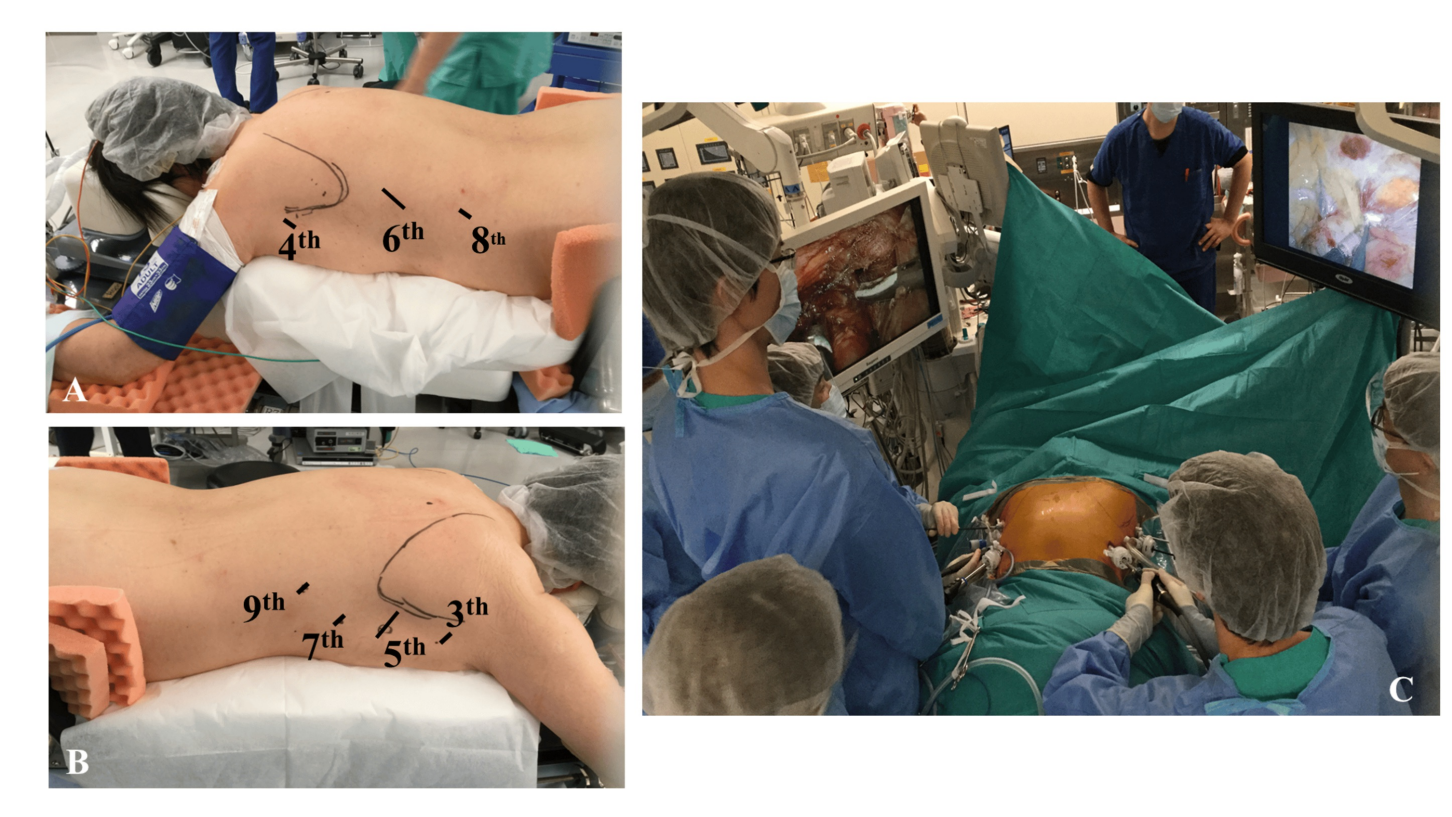
Surgical position Surgery was performed with three and four ports on the left and right sides, respectively. The surgery was conducted while simultaneously examining the images facing each other on the left and right sides.

In the prone position (Figure 2, Panels A and B) and under one-lung ventilation, we performed bilateral simultaneous VATS (Figure 2, Panel C). We initially approached from the right side with left one-lung ventilation and CO_2_ insufflation into the right thoracic cavity.

**Figure 3:**
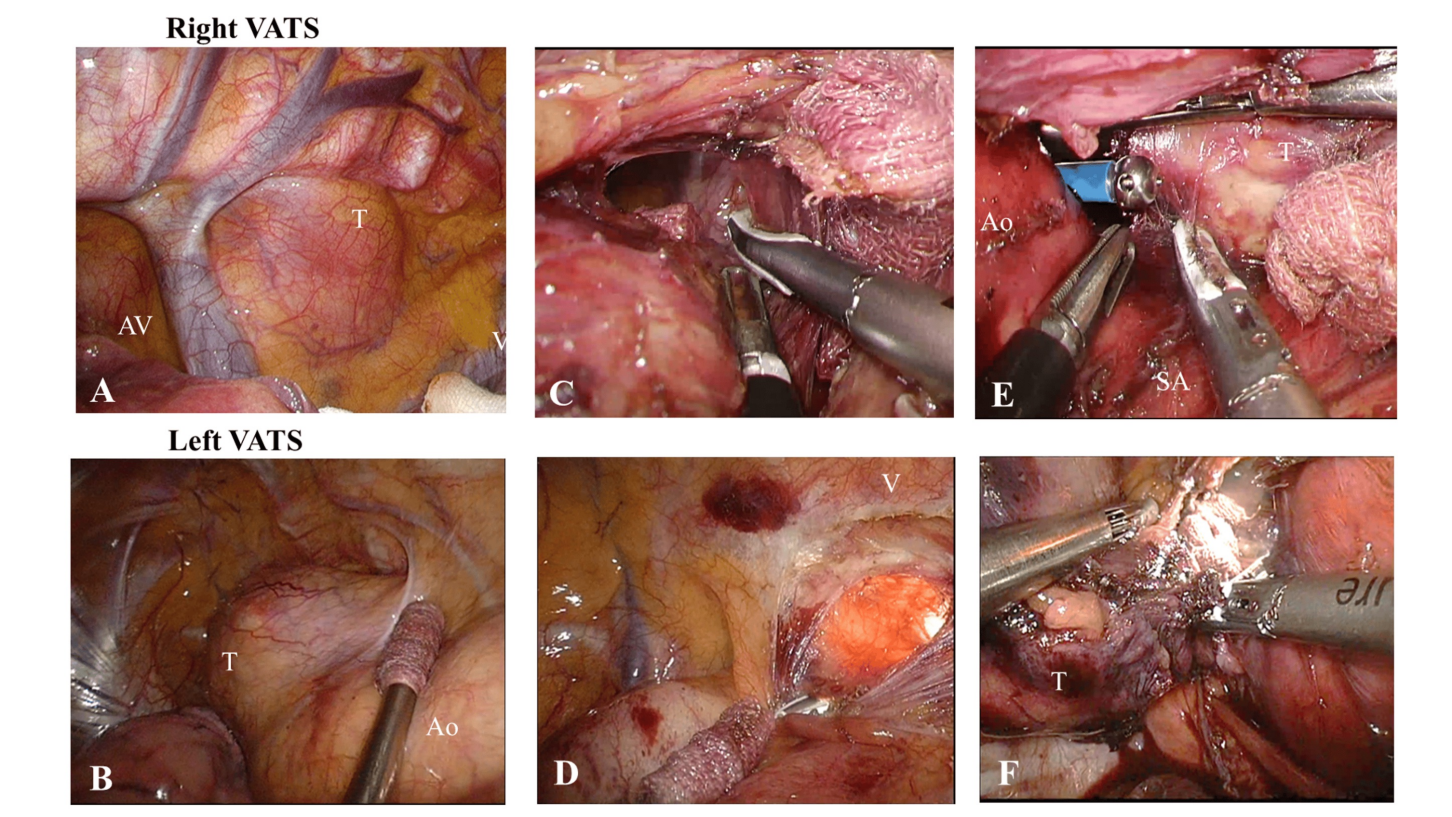
Surgical findings The mass observed in bilateral thoracic cavities (A, B). Opening of both thoracic cavities anterior to the vertebrae (C, D). Assistance of the right thoracic cavity operation from the left thoracic cavity and dissection around the subclavian artery (E). Tumor removal from the left thoracic cavity (F). Ao: Aorta; AV: Azygos vein; SA: Subclavian artery; T: Tumor; V: Vertebrae; VATS: Video-assisted thoracoscopic surgery.

The tumor was easily identified (Figure 3, Panel A), and we disconnected the azygos vein and dissected the mass from the esophagus and trachea. For the tumor dissection reaching the left thoracic cavity, we began our approach from the left side. While insufflating CO_2_ into the right thoracic cavity, we inserted a port into the left thoracic cavity for right one-lung ventilation and insufflated CO_2_ into the left thoracic cavity for intrathoracic observation. The tumor was easily identified (Figure 3, Panel B); the mediastinal pleura in front of the vertebrae was incised, and the tumor was dissected from the vertebrae to communicate with the right thoracic cavity (Figure 3, Panels C and D). The recurrent nerve was identified by dissection from the right; then the right side of the aorta was dissected, and the head side of the aorta was dissected from the left. This allowed communication between the left and right sides at the aortic level. Despite the presence of some adhesion around the blood vessels, no infiltration was observed. Hence, it was possible to dissect the vessels. Finally, the dorsal aspect of the left subclavian artery was dissected (Figure 3, Panel E), and the specimen was extracted (Figure 3, Panel F). The operative duration was 226 min, and there was a small amount of blood loss during the procedure.

**Figure 4:**
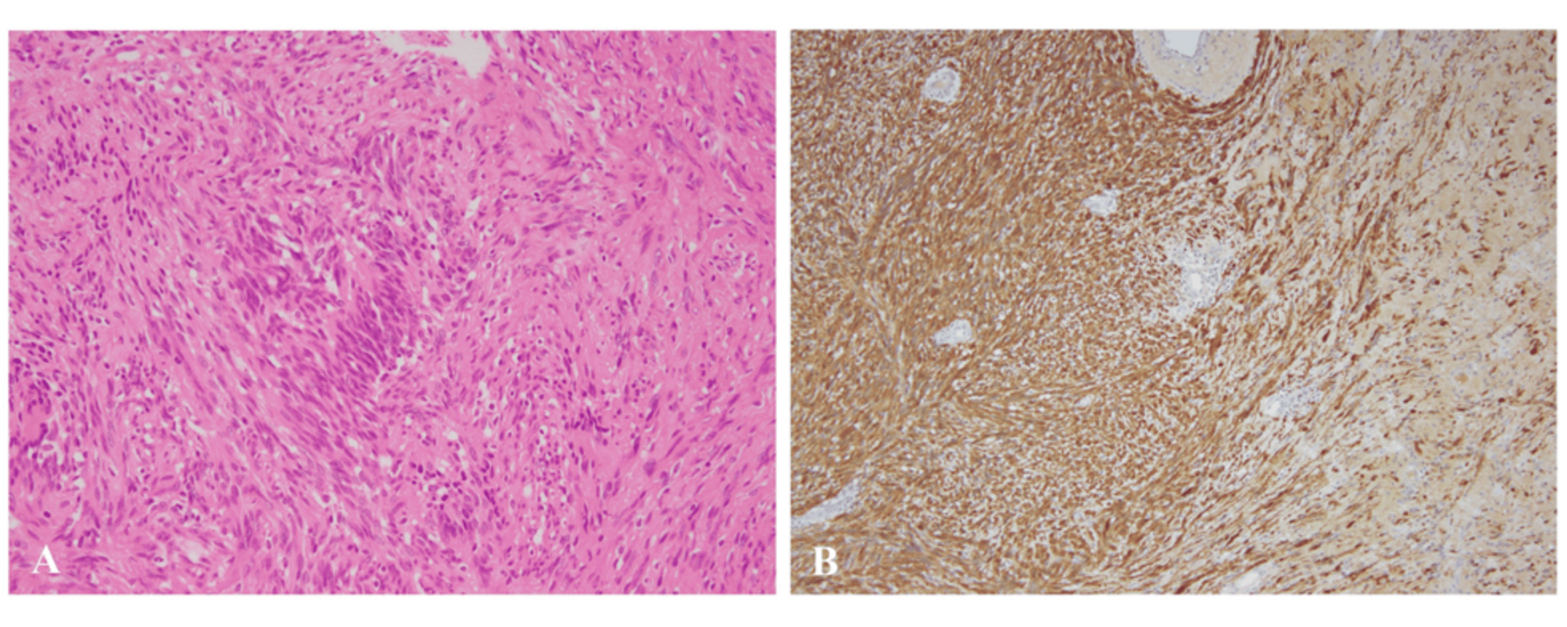
Pathological findings The area was covered with a fibrous capsule and showed a complex proliferation of spindle cells (A, hematoxylin-and-eosin staining; magnification, ×100). Immunostaining with S-100 showed diffuse positive results for spindle cells (B, magnification, ×100).

The patient developed chylothorax after surgery. She was fasted and underwent pleurodesis; the chest drain was removed after 28 days. Nonetheless, there were no other neurological symptoms, such as recurrent laryngeal nerve paralysis. The patient was discharged on postoperative day 39. The pathological diagnosis was schwannoma without malignant findings (Figure 4).

## Discussion

The occurrence of neurogenic tumors in the mediastinum is relatively rare. Neurofibromas and schwannomas are the most common types of neurogenic tumors [1], which mainly occur in the posterior mediastinum; these tumors rarely occur in the middle mediastinum. Most of these tumors originate from the intercostal nerves or sympathetic trunks [2], but they may also originate from the vagus nerve or recurrent laryngeal nerve [3]. Neurogenic tumors are typically benign, do not show clinical symptoms at the time of examination, and are often discovered incidentally through chest X-ray examinations [2]. On CT images, schwannomas appear as round or oval masses with clear boundaries. Low SI on T1-WI and heterogeneously high SI on T2-WI are characteristic findings in magnetic resonance imaging [4]. In the present case, the images suggested the presence of a neurogenic tumor. However, FDG accumulation was observed in the lesion on PET-CT. Although FDG accumulation can be observed in benign schwannomas [5], it was difficult to determine whether it was benign or malignant in this case.

The management of mediastinal schwannomas is determined based on several factors, including clinical findings and symptoms, tumor size, location, complications due to tumor growth, and pathology [6]. In general, schwannomas tend to grow gradually, and small, asymptomatic mediastinal schwannomas can be monitored for progression by serial imaging [7]. However, previous cases have described large schwannomas located adjacent to other tissues. There are also reported cases of malignant schwannomas infiltrating the thoracic aorta, leading to pathological aortic rupture and devastating hemorrhage [8].

In this case, the diagnosis (i.e., benign or malignant tumor) was not confirmed preoperatively; however, follow-up observation highlighted the possibility of respiratory distress due to tracheal compression in the future. Therefore, the possibility of aortic invasion in the case of malignancy could not be ruled out; hence, diagnostic treatment was selected.

Surgical resection is considered the optimal treatment option for neurogenic tumors [9]. Nevertheless, the selection of minimally invasive or open surgery depends on the location of the tumor and the tissue involved [10]. Mediastinal tumors, including schwannomas, can often be approached using minimally invasive or robotic surgery [11].

In the present case, the selection of the surgical approach was challenging. The head of the lesion was positioned to the left. However, it was believed that dissecting the lesion from the deep part of the aorta to near the right thoracic cavity with the right lateral position and left thoracic approach (which is typically performed in thoracic surgery) would be difficult. A previous report described thoracoscopic resection using a right thoracic approach for a large schwannoma located in the right mediastinum and in contact with the aorta [12]. In this patient, the tumor was located behind the trachea, where the esophagus is normally located. Therefore, we considered a right thoracic approach in the prone position, which is usually performed in esophageal surgery. With this approach, there was a possibility that the visibility of the deepest parts of the aorta, the left subclavian artery, and the left edge of the vertebrae might be insufficient. In addition, the possibility of malignancy could not be ruled out, and information from the left thoracic cavity was also necessary to determine whether it could be dissected from the major blood vessels. Consequently, we decided to adopt a simultaneous bilateral approach. As a bilateral approach is difficult with robotic surgery, we selected the VATS approach.

By performing surgical procedures from both sides simultaneously, deep anatomy can be viewed from the opposite side. Furthermore, effective movement of the lesion allows for efficient surgery with a good field of view and ensures safety. However, it was regrettable that the thoracic duct was damaged as a result, causing the chylothorax. During surgery, we were able to easily dissect the tumor from the posterior aspect of the trachea and the anterior aspect of the vertebral body via the right thoracic cavity. We also dissected the tumor from the head of the aorta and the anterior aspect of the vertebral body via the left thoracic cavity. As a result, we were able to confirm the positional relationship between the tumor and the surrounding tissues from both thoracic cavities. We believe that the bilateral simultaneous approach enabled the safe dissection of the tumor from the aorta and the left subclavian artery. In the present case, both the left and right vagus nerves and recurrent laryngeal nerves were identified and preserved, although the origin of the schwannoma was unclear.

This is a rare report of surgery using a bilateral simultaneous approach for schwannoma. This method requires the intervention of an esophageal surgeon. Moreover, depending on the presence or absence of vascular invasion, intraoperative intervention of a vascular surgeon may be necessary. In addition, there are several aspects to consider, such as compression of the airway from the dorsal side by the prone position during general anesthesia and reduced blood flow to the vertebral artery due to compression of the left subclavian artery. A preoperative plan for optimal surgical resection was devised by thorough preoperative evaluation and a multidisciplinary approach (i.e., anesthesiology, neurosurgery, and cardiovascular surgery).

## Conclusions

We report a case in which a relatively large tumor located in the middle mediastinum was resected using bilateral simultaneous VATS in the prone position. This approach shortens the operation time and allows for safer and more efficient dissection. Furthermore, it confirms the anatomy of the deep part of the surgical field from the other side during surgical field development. Thus, this approach offers numerous other advantages.
